# Chronic cough in post-COVID syndrome: Laryngeal electromyography findings in vagus nerve neuropathy

**DOI:** 10.1371/journal.pone.0283758

**Published:** 2023-03-30

**Authors:** Patricia García-Vicente, Antonio Rodríguez-Valiente, Carmen Górriz Gil, Reyes Márquez Altemir, Francisco Martínez-Pérez, Luis Fernando López-Pajaro, Jose Ramón García-Berrocal

**Affiliations:** 1 Department of Otolaryngology and Head and Neck surgery, Division of Neuro-Laryngology, Voice, and Swallowing Disorders, Puerta de Hierro Majadahonda University Hospital, Universidad Autónoma de Madrid, IDIPHISA, Madrid, Spain; 2 Department of Neurophysiology, Puerta de Hierro Majadahonda University Hospital, Universidad Autonoma de Madrid, IDIPHISA, Madrid, Spain; University of Catania, ITALY

## Abstract

**Background:**

Despite being a new entity, there is a large amount of information on the characteristics of SARS-CoV-2 infection and the symptoms of the acute phase; however, there are still many unknowns about the clinical features and pathophysiology of post-COVID syndrome. Refractory chronic cough is one of the most prevalent symptoms and carries both a medical problem and a social stigma. Many recent studies have highlighted the role of SARS-CoV-2 neurotropism, but no studies have demonstrated vagus nerve neuropathy as a cause of persistent chronic cough or other COVID-19 long-term effects.

**Objective:**

The main objective was to assess the involvement of the vagus nerve neuropathy as a cause of chronic cough and other post-COVID syndrome symptoms.

**Material and methods:**

This was a single-center observational study with prospective clinical data collected from 38 patients with chronic cough and post-COVID-19 syndrome. Clinical characteristics and laryngeal electromyographic findings were analyzed.

**Results:**

Clinical data from 38 patients with chronic cough after 12 weeks of the acute phase of COVID-19 infection were analyzed. Of these patients, 81.6% suffered from other post-COVID conditions and, 73.6% reported fluctuating evolution of symptoms. Laryngeal electromyography (LEMG) of the thyroarytenoid (TA) muscles and cricothyroid (CT) muscles was pathological in 76.3% of the patients. Of the patients with abnormal LEMG, chronic denervation was the most frequent finding (82.8%), 10.3% presented acute denervation signs, and 6.9% presented myopathic pattern in LEMG.

**Conclusions:**

LEMG studies suggest the existence of postviral vagus nerve neuropathy after SARS-CoV-2 infection that could explain chronic cough in post-COVID syndrome.

## Introduction

Coronavirus disease 2019 (COVID-19 or COVID) is an infection caused by severe acute respiratory syndrome coronavirus 2 (SARS-CoV-2). Although it is a novel entity, there is sufficient scientific evidence to confirm that the most prevalent acute phase symptoms are fever, cough, fatigue, and loss of taste and smell; especially during the first waves of the COVID pandemic [1]. In the acute phase of the disease, cough is a key symptom for the diagnosis, and it is also one of the most frequent complaints in the post-COVID syndrome or Long COVID (LC) [2,3].

Post-COVID syndrome is defined as the multiorgan symptom complex that affects those patients who remain symptomatic after 4–12 weeks of the acute phase of the disease [4,5]. Currently, this entity affects many people worldwide and has a great health and social impact during the pandemic. Although the exact prevalence of LC varies among studies, it is estimated to be present in at least 10% of all those infected, even reaching 20% in some recent studies [6,7]. Post-COVID cough is a disabling disorder for many patients. The severity depends on the intensity, duration, and association with concomitant symptoms. Currently, persistent cough carries a medical problem as well as a social stigma since it is associated with the possibility of contagious respiratory diseases [3].

Chronic cough is defined as a cough that lasts for eight weeks or longer, and the most common causes are upper airway cough syndrome, gastroesophageal reflux disease, asthma, and infection [8–10]. Nevertheless, another reason might explain cough in LC patients. Certain characteristics suggest a neurological etiology for cough, such as nonproductive cough; long duration of the complaint; and lack of response to antibiotics; asthma or reflux medication [8]. In addition, post-COVID cough is often associated with other pharyngolaryngeal and dysautonomic symptoms, which could be explained by the existence of a vagus nerve dysfunction [10].

Many studies have reported the role of virus neurotropism in the etiopathogenesis and pathophysiology of COVID-19 [11]. Multiple mechanisms may underlie the development of neurological manifestations, including hypoxia, hypercoagulability, endothelial dysfunction, general critical illness, inflammatory response, and neurotropism of SARS-CoV-2 [3,12,13]. Song et al. hypothesized that the pathways of neurotropism, neuroinflammation, and neuroimmunomodulation through the vagal sensory nerves might lead to a cough hypersensitivity state [3]. However, the intrinsic mechanism of neurogenic chronic cough is not well understood [3,11].

Several studies have revealed that angiotensin-converting enzyme 2 (ACE2) receptors and proteases such as transmembrane serine protease 2 (TMPRSS2) and furin are important for SARS-CoV-2 entry into host cells, since it is expressed in the neurons and glial cells [14]. Nevertheless, it is not known whether vagal neurons express ACE2 or TMPRSS2 or can be infected by SARS-CoV-2 [3,15].

Although many reports have illustrated the occurrence of cranial neuropathies in COVID-19, the injury mechanisms remain unclear [16,17]. A virus-mediated etiology is implicated in other head and neck conditions, including facial paralysis, Bell’s palsy, trigeminal neuralgia, glossopharyngeal neuralgia, and sudden sensorineural hearing loss. Associations with other viruses, such as herpes simplex virus, varicella-zoster, cytomegalovirus, influenza virus and human immunodeficiency virus have been documented [8].

Post-COVID vagus nerve neuropathy has already been reported. Rapoport et al. published a case series of 16 patients with dysphonia secondary to acute vocal fold paralysis after COVID-19 infection. Four patients (25%) agreed to undergo LEMG, and neuropathy was diagnosed in 100% of these patients. In most patients, they found other airway complaints, such as sore throat, excessive mucus, throat clearing, and cough, after COVID-19 recovery [18].

Postviral vagus nerve neuropathy may involve either sensory or motor branches. Laryngeal sensation is mediated by the superior laryngeal nerve, which also innervates the cricothyroid muscle. Dysfunction of this nerve can cause chronic cough and dysesthesias of the larynx, such as globus pharyngis, throat clearing or a tickle sensation. Motor branch impairment can lead to vocal fatigue, dysphonia, vocal fold paresis, or swallowing difficulties.

Since it is fundamentally a sensory dysfunction, it is often difficult to make a clinical evaluation and diagnosis. It is known that a sensory deficit can be inferred from the presence of a motor deficit, and fiberoptic endoscopy can be helpful to establish a diagnosis [8]. For instance, the inability to lengthen a vocal fold has long been claimed to reveal a superior laryngeal nerve neuropathy [8]. Fiberoptic endoscopic evaluation of swallowing with sensory testing allows the calculation of laryngopharyngeal sensitivity based on the air pulse required to stimulate a laryngeal adductor reflex [12]. Nevertheless, many patients with chronic neurogenic cough may have a normal macroscopic and functional appearance of the larynx. Other techniques that should be considered include laryngeal electromyography evaluation and surface-evoked laryngeal sensory action potentials to detect conduction along the internal branch of the superior laryngeal nerve [10].

The objective of the present study was to assess the involvement of the vagus nerve neuropathy as a cause of chronic cough and other post-COVID syndrome symptoms.

## Materials and methods

This was a single-center, descriptive observational study with prospective clinical data collection. The inclusion criteria included patients older than 18 years presenting with chronic cough 12 weeks after the diagnosis of acute phase of COVID-19 infection as confirmed by a reverse transcriptase-polymerase chain reaction (RT‒PCR) assay of a nasopharyngeal swab sample. Only those infected by virus strains prior to Omicron and Delta variants and who denied having chronic cough prior to COVID-19 were included.

The recruitment dates were from January 2021 to November 2021. This study was approved by the Ethical Committee of Clinical Research from Puerta de Hierro Majadahonda University Hospital (PI 227/19). All patients provided written informed consent to be included in the study (S1 Appendix).

A complete medical history was carried out where epidemiological data of the sample were collected. Data included the characteristics of acute COVID-19 infection severity (using the Brescia-COVID Respiratory Severity Scale [19], hospitalization admission need and acute phase symptoms), post-COVID conditions related to the otorhinolaryngology area (dysphonia, dysphagia, laryngeal paresthesia, laryngospasm, hyposmia/dysgeusia, odynophagia, vertigo, hearing loss, and tinnitus) and other persistent symptoms (neurological, dysautonomic, respiratory, and musculoskeletal disorders).

A complete otolaryngologist examination was performed in all cases, comprising bilateral otoscopy, anterior rhinoscopy, oropharyngoscopy, flexible video laryngoscopy and stroboscopy to analyze the correct symmetry and presence of mucosal wave in phonation. General inspection of the supraglottic structures and the vocal folds were examined. Motor activity was examined both at rest and during phonation, analyzing laryngeal symmetry, aberrant muscle hyperfunction or hypofunction, tremor, motility of the vocal cords, dysdiadokinesias, fibrillations, and synkinesis.

The electrodiagnostic study consisted of a coaxial concentric needle laryngeal electromyography (LEMG) examination using a Synergy Electromyographs Version 15.0 (Viasys Healthcare UK Ltd). It was carried out with pass bands of 3 Hz to 3 kHz and a 50 Hz notch filter, with a 100 ms sweep and variable amplitude from 100 mV. Data were then recorded digitally for off-line analysis.

LEMG studies were carried out in office by otorhinolaryngology and neurophysiology specialists and performed under intralaryngeal anesthesia. While the patient was in the sitting position with the neck sightly extended, the larynx was anaesthetized by injecting 1 cc of 5% lidocaine through the cricothyroid membrane. A 37 mm concentric needle electrode was used to test the thyroarytenoid (TA) muscle. It was inserted into the skin at the midline of the upper edge of the cricoid arch through the cricothyroid membrane in most of the patients; only in some cases was it introduced through the thyrohyoid membrane. A 25 mm needle was used to test the cricothyroid (CT) muscle. It was pierced through the cricothyroid notch, 5 mm off midline, 50° laterally, 15° superiorly, and introduced approximately 15–20 mm.

CT and TA muscle activation were examined bilaterally. The patients were asked to pronounce the vowel /i:/ repeatedly after inserting the needle until MUAPs (motor unit action potentials) were detected on the screen. The patient was asked to raise the pitch of their voice to examine the CT muscle. The presence or absence of spontaneous activity was assessed. The amplitude and duration of MUAPs, the presence of polyphasic potentials and the recruitment pattern were also tested. These items allowed establishment of the etiology of the injury (neurogenic, myopathic) as well as its evolutionary state (acute, subacute, chronic lesion).

## Results

The demographic and clinical characteristics of the sample are shown in Table 1. In total, 38 patients were included in the study; 24 (63.2%) were females,14 (36.8%) were males, and the median age was 57 years old (range 32–85 years). Regarding personal medical history, 13 patients (34.2%) had a previous diagnosis of gastroesophageal reflux disease, of which 77% were taking proton pump inhibitors, 3 patients (7.9%) suffered from well-controlled asthma and 1 (2.6%) had mild chronic obstructive pulmonary disease. None of them presented chronic cough prior to COVID-19 diagnosis.

**Table 1:** Summary of clinical characteristics of the sample.

Characteristic	Number (n)	Percent of Cohort (%)
**Sex**		
Male	14	36.8
Female	24	63.2
**Age (years)**		
18–40	2	5.3
41–60	21	55.3
61–80	14	36.8
>80	1	2.6
**ACE Inhibitors /ARA-II**	3	7.9
**CVRF**		
Diabetes Mellitus	4	10.5
Dislipemia	9	23.7
Arterial hypertension	9	23.7
No CVRF	24	63.2
**Gastroesophageal reflux disease (GERD)**		
GERD with PPIs treatment	10	26.3
GERD without treatment	3	7.8
No GERD	25	65.8
**Respiratory disorders**		
Asthma	3	7.9
COPD	1	2.6
No respiratory disorders associated	34	89.5
**Smoking status**		
Smoker	7	18.4
Non-smoker	31	81.6

***ACE*:** *Angiotensin-converting enzyme*. ***ARA-II***: *Angiotensin II receptor antagonists*. ***CVRF***: *Cardiovascular risk factors*. ***PPIs***: *Proton Pump Inhibitors*. ***COPD***: *Chronic obstructive pulmonary disease*.

The Brescia-COVID respiratory severity scale [19] is a stepwise management approach for COVID-19 patients based on clinical severity. The severity was mild in the majority of the sample (Table 2). Only 6 patients (15.6%) needed hospital admission, with a median stay duration of 7 days. The most prevalent acute phase symptom was fever (89.5%), followed by nonproductive cough (84.2%), myalgia (73.7%) and dyspnea (34.2%). Only 8 patients (21.2%) presented with taste and smell disorders.

**Table 2:** Acute phase of COVID-19 infection.

Characteristic	Number (n)	Percent of Cohort (%)
**Severity (BCRSS** ^**a**^ **)**		
Grade 0	22	57.9
Grade 1	10	26.3
Grade 2	2	2.3
Grade 3	4	10.5
**Admission**		
Hospitalization ward	4	10.5
Intensive Care Unit	2	5.3
No hospital admission	31	81.6
**Main acute phase symptoms**		
Fever	34	89.5
Cough	32	84.2
Myalgia	28	73.7
Dyspnea	13	34.2
Headache	12	31.6
Odynophagia	3	7.9
Taste and smell disorders	8	21.1

^a^**BCRSS**: Brescia-COVID Respiratory Severity Scale.

All included patients presented with persistent chronic cough after 12 weeks of the acute phase of COVID-19 infection, and 81.6% suffered from other post-COVID conditions, as detailed in Table 3. A total of 73.6% mentioned a fluctuating course of symptomatology. Concerning those related to vagus nerve neuropathy symptoms, the most prevalent was dysphonia (68.8%), followed by globus pharyngis (56.3%) which was described as a foreign body sensation, laryngospasm (10.5%), and odynophagia (10.5%). It is worth highlighting the presence of fatigue (63,2%), arthromyalgias (39.5%), memory loss (10.5%), and attention issues (18.4%), paresthesia (10.5%), and dysautonomic symptoms, such as palpitations, arterial hypotension, and gastrointestinal disorders, in 23.7% of the patients.

**Table 3:** Long-term symptoms associated with post-COVID syndrome.

Symptom	Number (n)	Percent of Cohort (%)
**Chronic cough**	38	100
**ENT symptoms**		
Dysphonia	24	63.2
Globus pharynges	20	56.3
Laryngospam	4	10.5
Odynophagia	4	10.5
Dysphagia	4	10.5
Taste and smell disorders	4	10.5
Hearing loss, tinnitus	3	7.84
Vertigo	1	2.63
**Neurologic symptoms**		
Fatigue	24	63.2
Dysautonomic symptoms	9	23.7
Memory loss, brain fog, attention disorders	7	18.4
Headache	5	13.2
Paresthesias	4	10.5
**Respiratory symptoms**		
Dyspnea	6	15.8
Thoracic pain	6	15.8
**Musculoskeletal symptoms**		
Arthromyalgias	15	39.5

Nasofibrolaryngoscopy and stroboscopy were normal in 68.4% of the sample. However, 31.6% of the patients presented laryngeal signs suggestive of hypoesthesia, such as the absence of a cough reflex when touching supraglottic structures or salivary retention. Vocal cord motility was preserved in 94.7%, and only two patients had a unilateral vocal cord paralysis.

LEMG of the TA muscles and CT muscles (Tables 4 and S1) was normal in 23.7% of the cohort. Among patients with abnormal LEMG (76.3%), chronic denervation was the most common finding (82.8%), 10.3% had acute denervation signs, and 6.9% showed a myopathic pattern in LEMG. Only one patient had concomitant chronic neuropathy and myopathy. LEMG could not be completed in 12 out of the 152 muscles tested due to poor tolerance or anatomical difficulties.

**Table 4:** Electromyographic findings.

LEMG finding	Number (n)	Percent of Cohort (%)
**Normal**	9	23.7
**Pathological**	29	76.3
Chronic denervation pattern	24	63.2
Acute denervation pattern	3	7.9
Myopathic pattern	2	5.3

*Detailed data can be found in* ***S1 Table*.**

The details of comorbidities and symptoms associated with the participants who had abnormal LEMG findings can be found in S2 Table. Among the results, 72.4% of patients had another systemic symptom. Fatigue was the most prevalent, present in 65.5%. They had another associated laryngeal symptom in 96.6% of the cases. Only one patient had chronic cough without other otorhinolaryngologic symptoms, but suffered from many systemic symptoms such as fatigue, headache, brain fog, attention disorders and arthromyalgias.

## Discussion

At the beginning of the pandemic, COVID-19 was predominantly defined as a lower respiratory tract illness characterized by bilateral interstitial pneumonia. However, several investigations have been carried out, demonstrating neurological symptoms linked to COVID-19 infection [11,12,16,20].

Regarding peripheral nervous system effects, hyposmia and dysgeusia are the most common complaints as an acute phase symptom as well as conforming to post-COVID syndrome [1,21]. Other frequent manifestations are fatigue, joint pain, cough, dyspnea, palpitations, chest pain, nausea, diarrhea, depression, sleeping difficulties, concentration problems, dizziness, and headache [1,5,22]. The prevalence of these symptoms differs among different authors. In a recent review, Boaventura et al. concluded that the most frequent symptom of post-COVID conditions is fatigue, which is independent of the acute disease severity or the presence of respiratory problems. In our cohort, fatigue was also the most prevalent systemic symptom reported. It was present in 63.2% of the patients.

Chronic cough is one of the most frequent post-COVID symptoms. The prevalence of cough differs across studies (9.9–20%) [23,24]. Cough is frequently considered as a respiratory sequela but is often not found in relation to pulmonary problems, pneumonitis, or acute-phase pneumonia, and the physical examination is frequently normal [3,25]. In our cohort, 84.2% presented with a mild infection in the acute phase, and 15.8% of the patients did not present with cough in the acute phase. The absence of pulmonary sequelae in these patients and the fact that most of them did not have severe pneumonia in the acute phase led us to consider the existence of cough reflex impairment after COVID-19.

**Fig 1:**
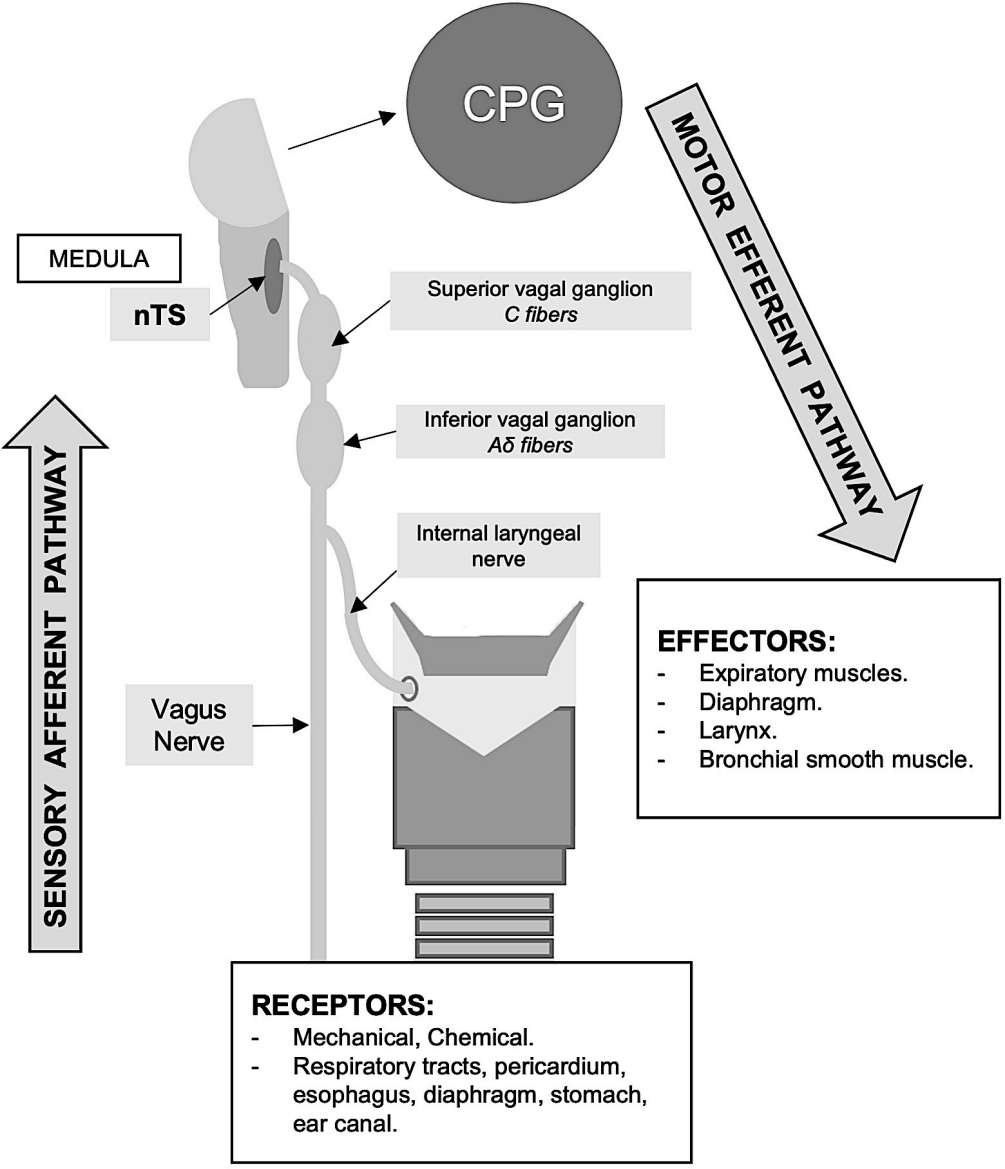
The cough reflex arc. nTS: Nucleus tractus solitarius. CPG: Central pattern generator.

The cough reflex arc is made up of three main pathways, the sensory afferent pathway, central pathway, and motor efferent pathway, as shown in Fig 1. Airway sensory nerves activated in response to a stimulus travel through the internal laryngeal nerve, the superior laryngeal nerve, and vagus nerve to the medulla and terminate in the nucleus tractus solitarius (nTS). The respiratory central pattern generator (CPG) modifies the activity of the inspiratory and expiratory muscles and leads to cough. C-fibers, with cell bodies in the superior vagal (jugular) ganglion, play a role in facilitating nTS activity, and A δ-fibers, with cell bodies in the inferior vagal (nodosum) ganglion, are the main vagal fibers mediating cough [14,26].

In our study LEMG was pathological in 76.3% of the patients. These findings and the absence of other possible causes of cough suggest the existence of a vagus nerve neuropathy as responsible for chronic cough and symptoms of laryngeal irritability after COVID-19.

Classically, laryngeal irritability symptoms were included in the Irritable Laryngeal Syndrome introduced as laryngeal hyperresponsiveness triggered by exposure to noxious stimuli such as allergens, gastroesophageal reflux, or toxins [27].

Recently, a new paradigm has been proposed to describe this entity, the cough hypersensitivity syndrome, which is defined as a clinical entity characterized by cough frequently induced by low levels of exposure to thermal, mechanical, or chemical stimuli [28]. The main mechanism of this hypersensitivity lies in dysregulated sensory neural pathways and in the central process of cough regulation itself. Respiratory viruses, such as SARS-CoV-2, infect and replicate in airway epithelial cells, causing proinflammatory responses in the host and the production of soluble neurotrophic factors. They can also infect sensory neurons and produce neuronal phenotypic changes [29–31].

The mechanisms of the Cough Hypersensitivity Syndrome neural dysregulation are not exactly known. The fact that LEMG shown signs of active and chronic denervation of the intrinsic laryngeal muscles may suggest the existence of a laryngeal nerve dysfunction rather than the presence of central dysregulation of the cough reflex [31].

In our cohort, 84.4% presented with additional laryngeal symptoms other than chronic cough, prevalence rises in those with abnormal LEMG (96.6%). The most prevalent symptom was dysphonia (63,2%), but it is relevant to point out that the vast majority had normal vocal fold mobility, with only two (5.3%) patients presenting with vocal fold paralysis.

Most patients had fluctuating dysphonia, which often worsened during the day. The dysphonia could be explained by a mild impairment of the vagal motor branch, especially in the patients with pathological LEMG of the muscle TA. However, it is important to note that it could also be a secondary to a muscle tension dysphonia. Vertigan et al. have conducted numerous studies in recent years demonstrating an overlap between chronic refractory cough and other laryngeal disorders, particularly vocal cord dysfunction and muscle tension dysphonia [32,33].

In addition, the vagus nerve also controls certain body functions, such as digestion, heart rate, and other parasympathetic functions. In 44.7% of patients, we found concomitant dysautonomic symptoms such as rapid or slow heart rate, anxiety, fatigue, and gastrointestinal symptoms. The prevalence of these symptoms was higher in those patients with abnormal LEMG.

Song et al. published the hypothesis that post-COVID syndrome could be explained by a generalized neuronal hypersensitivity. Their study emphasized the fact that cough is frequently associated with other symptoms, including chronic fatigue, dyspnea, chronic pain, and cognitive impairment [3]. These results correspond to what was found in our sample, where more than half of the patients had other associated neurological symptoms.

Post-COVID cough can become a very disabling disorder for many patients. Since cough episodes can occur at any time, even in relation to speech, it can often lead to work and social disability. In addition, many patients often undergo numerous conventional diagnostic tests without a diagnosis and receive repeated treatments without clinical improvement.

Considering the results of our study, it is important to consider the role of the larynx and vagus nerve as a target of treatment [33]. In the last decades, numerous studies have been conducted on chronic neurogenic cough, which included various alternatives such as speech pathology treatment, neuromodulators, botulinum toxin A injection in TA muscles or superior laryngeal nerve blockade [33–36].

Speech pathology treatment increases voluntary control over cough to prevent or interrupt cough episodes [34,35]. Centrally acting neuromodulators such as gabapentin and amitriptyline decrease cough frequency and cough quality of life [33]. Recently, Simpson et al. reported their experience with blockade of the internal branch of the superior laryngeal nerve in patients with chronic neurogenic cough attributed to laryngeal hypersensitivity. They were successful in reducing the cough severity index, from a pretreatment average of 26.8 to 14.6 posttreatment (p < 0.0001) [37].

Further studies are still needed to determine which of the above treatments is best for these patients. The main limitations are the difficulty of obtaining a homogeneous sample and the possibility of measuring response to treatment objectively. The results of the present study support the idea of the existence of a vagus nerve dysfunction after COVID-19. For this reason, treatments focused on the vagus nerve such as nerve blockade may have a more significant role compared to centrally acting neuromodulators.

Although LEMG is not widely available, it is important to consider the existence of chronic neurogenic cough after COVID-19 to provide the correct diagnosis and the most appropriate treatment to patients.

## Conclusions

Postviral vagus nerve neuropathy is a relevant entity that could explain chronic cough in post-COVID syndrome. Since it is principally a sensory deficit, clinical evaluation and diagnosis are challenging and often made by exclusion. ENT and neurophysiologic studies can help us diagnose chronic neurogenic cough by confirming the presence of vagus nerve neuropathy. Therefore, it is important to suspect this entity in patients with unexplained chronic cough to propose a correct treatment in which speech pathology, blockade of the superior laryngeal nerve and neuromodulators could play an essential role.

## Limitations

This study was a preliminary investigation. Our results support the existence of vagus nerve neuropathy in patients with chronic cough after COVID -19. However, further studies are needed to better understand this entity and the utility of LEMG in vagal neuropathy. Despite these limitations, the goal of this study was to provide direction for future research on vagal neuropathy after COVID -19.

## Supporting information

S1 Appendix Informed consent form. (DOCX)

S1 Table Electromyographic findings. (DOCX)

S2 Table Details of comorbidities and symptoms of patients with abnormal LEMG. (DOCX)

S1 Dataset Research data. (XLSX)
