# Supplementary material for: Chronic cough in post-COVID syndrome: Laryngeal electromyography findings in vagus nerve neuropathy
Source: PLoS One. 2023 Mar 30;18(3):e0283758. doi: 10.1371/journal.pone.0283758 (PMC10062549; doi:10.1371/journal.pone.0283758)
Supplement: S1 Appendix — (DOCX) [file pone.0283758.s001.docx]

**S1 APPENDIX - INFORMED CONSENT FORM**

Patient Name

Clinical Identification Number

Date

This Informed Consent Form is for men and women who are invited to participate in the research on **“Laryngeal electromyography findings in vagus nerve neuropathy after COVID-19.”**

Principal Reseachers: Patricia García Vicente and Antonio Rodriguez Valiente. ENT Department, Puerta de Hierro Majadahonda University Hospital.

You have been invited to participate in a research project under the supervision of Dr. Patricia García Vicente and Dr. Antonio Rodriguez Valiente at Puerta de Hierro Majadahonda University Hospital. The purpose of this research is to assess the influence of vagal neuropathy in patients with post-COVID Syndrome.

1. Your participation includes:

a) Medical visits and follow-up telephone interviews. The information provided in the interview will be confidential.

b) The medical visits will be: first face-to-face consultation, consultation to perform neurophysiological studies, follow-up telephone consultation at 3 months, 6 months and 12 months. A Clinical History and an ENT examination will be carried out, which will be the same as that which would be carried out in the case of not being included in the research project.

c) Laryngeal Electromyography will be performed with the Neurophysiology service.

d) All data will be collected and statistically analyzed confidentially.

1. Participation is voluntary. You can leave the study at any time.
2. Therapeutic management will be according to your pathology.
3. Risks and side effects that may be associated the study:

a) Those inherent to neurophysiological tests, prior knowledge by informed consent.

b) Those inherent to otorhinolaryngological tests, prior knowledge by informed consent.

1. Participation in this research project does not replace or increases the number of visits or explorations that would be carried out if you were not included in the study.
2. If you wish, you will be informed of all the results of the investigation.

Name of Participant

- I HAVE RECEIVED INFORMATION about the research work.
- I UNDERSTAND the information received and have had the opportunity to ask questions and have been provided answers correctly.
- I GIVE CONSENT FREELY to participate in the research work

#### Madrid,

#### Date: (day/month/year)

Signature of Participant Signature of Researcher

**The study carried out has the approval of the Ethical Committee of Clinical Research from Hospital Puerta de Hierro Majadahonda (PI 227/19).**
