# Supplementary material for: Chronic cough in post-COVID syndrome: Laryngeal electromyography findings in vagus nerve neuropathy
Source: PLoS One. 2023 Mar 30;18(3):e0283758. doi: 10.1371/journal.pone.0283758 (PMC10062549; doi:10.1371/journal.pone.0283758)
Supplement: S1 Table — (DOCX) [file pone.0283758.s002.docx]

**S2 Table. Electromyographic findings.**

|  | | **Right CT** | **Left CT** | **Right TA** | **Left TA** | **Description** |
| --- | --- | --- | --- | --- | --- | --- |
|  | *Sp/Amp/Du/Polif/R* | | *Sp/Amp/Du/Polif/R* | *Sp/Amp/Du/Polif/R* | *Sp/Amp/Du/Polif/R* |  |
| 1 | N/2+/2+/N/IF | | N/2+/2+/N/IF | N/2+/2+/N/IF | N/2+/1+/N/IF dec | Chronic bilateral denervation of TA and CT. |
| 2 | N/N/N/N/IF | | N/1+/1+/N/IF | N/N/N/N/IF | N/1+/2+/N/IF dec | Chronic denervation of left TA and CT. |
| 3 | N/N/N/N/IF dec | | N/1+/1+/1+/IF dec | N/1-/N/2+/IF red | N/1+/1+/1+/IF dec | Myopathic pattern of right TA. Mild chronic denervation of left TA and CT. |
| 4 | NP | | N/N/N/N/IF | N/2+/2+/1+/IF | NP | Chronic denervation of right TA. |
| 5 | N/N/N/3+/IF | | N/N/N/3+/IF | N/N/N/3+/IF | N/N/N/3+/IF | Signs of reinnervation of both TA and CT. |
| 6 | N/N/N/N/IF | | N/N/N/N/IF | N/N/N/N/IF | N/N/N/N/IF | Normal. |
| 7 | N/1+/1+/1+/IF dec | | N/1+/1+/1+/IF dec | N/N/N/N/IF | N/1+/1+/1+/IF dec | Chronic bilateral denervation of both CT and left TA. |
| 8 | NP | | NP | 2+/N/N/N/IF | NP | Acute denervation of right TA. |
| 9 | N/1+/1+/1+/IF dec | | N/1+/1+/1+/IF dec | N/N/N/N/IF | N/N/N/N/IF | Chronic bilateral denervation of both CT. |
| 10 | N/1+/N/2+/IF | | N/N/N/2+/IF | N/1+/N/2+/IF | N/N/N/2+/IF | Bilateral myopathic pattern of both TA and CT. |
| 11 | N/N/N/N/IF | | N/N/N/N/IF | N/N/N/N/IF | N/N/N/N/IF | Normal. |
| 12 | N/N/N/N/IF | | N/N/N/N/IF | N/N/N/N/IF | N/N/N/N/IF | Normal. |
| 13 | N/N/N/N/IF | | N/N/N/N/IF | N/N/N/N/IF | N/N/N/N/IF | Normal. |
| 14 | N/+1/+1/+1/IF IM | | N/N/N/N/IF | N/N/N/N/IF | N/+1/+1/+1/IF dec | Chronic denervation of right CT and left TA. |
| 15 | N/N/N/N/IF | | N/+1/+1/+1/IF | N/N/N/N/IF | N/N/N/N/IF | Chronic denervation of left CT. |
| 16 | N/N/N/N/IF | | N/N/N/N/IF | N/+1/N/N/IF | N/+1/N/N/IF | Chronic bilateral denervation of both CT. |
| 17 | 2+/N/N/N/IF | | N/N/N/N/IF | 2+/N/N/N/IF | N/N/N/N/IF | Acute denervation of right CT and right TA. |
| 18 | NP | | N/N/N/3+/IF | N/N/N/N/IF | N/N/N/2+/IF | Signs of reinnervation of left CT and left TA . |
| 19 | N/N/N/N/IF | | N/N/N/3+/IF | N/N/N/N/IF | N/N/N/3+/IF | Signs of reinnervation of left CT and left TA. |
| 20 | N/N/N/N/IF | | N/N/N/N/IF | N/N/N/N/IF | N/N/N/N/IF | Normal. |
| 21 | N/1+/2+/1+/IF dec | | N/1+/1+/1+/IF dec | N/N/N/N/IF | N/N/N/N/IF | Chronic bilateral denervation of both CT. |
| 22 | N/N/N/N/IF | | N/N/N/N/IF | N/N/N/N/IF | N/N/N/N/IF | Normal. |
| 23 | N/N/N/3+/IF | | N/2+/2+/N/IF | N/2+/2+/N/IF | N/N/N/N/IF | Chronic denervation of left TA and CT and signs of reinnervation of right TA. |
| 24 | N/1+/2+/1+/IF dec | | N/1+/1+/1+/IF dec | N/N/N/N/IF | N/N/N/N/IF | Chronic bilateral denervation of both CT. |
| 25 | N/1+/2+/1+/IF dec | | N/1+/1+/1+/IF dec | N/N/N/N/IF | N/1+/2+/N/IF dec | Chronic bilateral denervation of both CT and left TA. |
| 26 | N/1+/1+/2+/IF | | N/1+/1+/1+/IF | N/N/N/N/IF | N/N/N/N/IF | Chronic bilateral denervation of both CT. |
| 27 | N/1+/2+/1+/IF red | | N/1+/2+/1+/IF red | NP | N/N/N/N/IF | Chronic bilateral denervation of both CT. |
| 28 | N/N/N/N/IF | | NP | N/N/N/N/IF | N/N/N/N/IF | Normal. |
| 29 | NP | | NP | NP | 2+/N/N/N/IF | Acute denervation of left TA. |
| 30 | N/+1/+1/+1/IF red | | N/N/N/N/IF | N/N/N/N/IF | N/+1/+1/+1/IM IF | Chronic denervation of right CT and left TA. |
| 31 | N/N/N/N/IF | | N/N/N/N/IF | N/N/N/N/IF | N/N/N/N/IF | Normal. |
| 32 | N/1+/1+/2+/IF | | N/1+/2+/1+/IF | N/N/N/N/IF | N/N/N/N/IF | Chronic bilateral denervation of both CT. |
| 33 | N/1+/N/2+/IF | | N/+1/N/2+/IF | N/1+/N/2+/IF | N/N/N/2+/IF | Bilateral myopathic pattern of both TA and CT. |
| 34 | N/N/N/N/IF | | N/N/N/N/IF | N/N/N/N/IF | N/N/N/N/IF | Normal. |
| 35 | N/1+ /2+/1+/IF dec | | N/1+/1+/1+/IF dec | NP | N/N/N/N/IF | Chronic bilateral denervation of both CT. |
| 36 | N/2+/2+/N/IF | | N/N/N/N/IF | N/1+/1+/2+/IF | N/1+/1+/1+/IF | Chronic denervation of right CT and both TA. |
| 37 | N/1+/1+/1+/IF dec | | N/1+/1+/1+/IF dec | N/N/N/N/IF | N/2+/2+/N/IM IF | Chronic bilateral denervation of CT and left TA. |
| 38 | N/N/N/N/IF | | N/1+/1+/N/IF | N/N/N/N/IF | N/1+/2+/N/IF dec | Chronic denervation of left TA and left CT. |
| **CT,** cricothyroid muscle; **TA,** thyroarytenoid muscle; **Sp,** spontaneus activity; **Polif,** poliphasia. **R,** recruitment pattern; **IF**, interference pattern; **N**, normal; **Dec,** decreased; **IM,** Intermediary; **NP**, not performed. | | | | | | |
