# Supplementary material for: Chronic cough in post-COVID syndrome: Laryngeal electromyography findings in vagus nerve neuropathy
Source: PLoS One. 2023 Mar 30;18(3):e0283758. doi: 10.1371/journal.pone.0283758 (PMC10062549; doi:10.1371/journal.pone.0283758)
Supplement: S2 Table — (DOCX) [file pone.0283758.s003.docx]

**S3 Table. Details of comorbidities and symptoms of patients with abnormal LEMG.**

| **Patient** | **Sex** | **Age** | **Comorbidities** | **Laryngeal symptoms** | **Other symptoms** |
| --- | --- | --- | --- | --- | --- |
| **1** | Female | 73 | COPD | Globus pharyngis, odynophagia | None |
| **2** | Female | 49 | GERD | Globus pharyngis | Fatigue, dyspnea, arthromyalgias |
| **3** | Male | 59 | AH, DL | Dysphonia | Fatigue, paresthesias, headache, brain fog, arthromyalgias |
| **4** | Male | 66 | AH, DM, Smoker | HL, tinnitus, vertigo, dysphonia | None |
| **5** | Female | 32 | None | Taste and smell disorders, dysphonia | Arthromyalgias, abdominal pain |
| **7** | Female | 60 | GERD, Asthma | Globus pharyngis, dysphonia | Fatigue |
| **8** | Male | 55 | GERD, Smoker | Taste and smell disorders, globus pharyngis | Fatigue, dysautonomia, thoracic pain, abdominal pain |
| **9** | Male | 63 | DL | HL, tinnitus, globus pharyngis, dysphonia | Fatigue, headache, brain fog, memory loss, arthromyalgias |
| **10** | Female | 49 | None | Laryngospasm, globus pharyngis, dysphagia | Fatigue, arthromyalgias |
| **14** | Female | 64 | DL | Taste and smell disorders, globus pharyngis, dysphonia | Fatigue, headache, insomnia, dyspnea, thoracic pain |
| **15** | Female | 42 | None | Globus pharyngis, dysphonia | Fatigue, arthromyalgias |
| **16** | Female | 54 | None | Laryngospasm, dysphagia | Fatigue, dysautonomia, thoracic pain |
| **17** | Female | 55 | GERD | Laryngospasm | None |
| **18** | Male | 45 | None | Globus pharyngis, dysphonia | None |
| **19** | Female | 55 | None | Dysphonia | Fatigue, brain fog, dysautonomia |
| **21** | Female | 58 | GERD | Globus pharyngis | Fatigue, paresthesias, arthromyalgias |
| **23** | Male | 55 | AH | Globus pharyngis, dysphonia | Fatigue, headache, thoracic pain |
| **24** | Female | 53 | GERD | None | Fatigue, headache, brain fog, attention disorders, arthromyalgias |
| **25** | Male | 61 | AH, DL, DM | Dysphonia, odynophagia | Dyspnea, thoracic pain |
| **26** | Male | 66 | AH | Globus pharyngis, dysphonia | None |
| **27** | Female | 42 | Asthma | Dysphagia, dysphonia | Fatigue |
| **29** | Male | 55 | GERD, Smoker | Taste and smell disorders, globus pharyngis | Fatigue, dysautonomia, dyspnea, thoracic pain |
| **30** | Female | 85 | AH, DL, Smoker | HL, tinnitus, dysphonia | None |
| **32** | Female | 61 | DL, Asthma | Globus pharyngis | Fatigue |
| **33** | Male | 49 | None | Laryngospasm | None |
| **35** | Female | 62 | DL | Dysphonia | Fatigue, paresthesias, brain fog, insomnia, arthromyalgias |
| **36** | Female | 50 | None | Dysphonia | None |
| **37** | Female | 63 | GERD | Globus pharyngis, dysphonia, odynophagia | Fatigue, dyspnea, |
| **38** | Female | 49 | None | Globus pharyngis | Fatigue, brain fog, urinary incontinence, |

***GERD****: Gastroesophageal reflux disease.* ***DM****: Diabetes Mellitus.* ***DL****: Dislipemia.* ***AH****: Arterial hypertension.* ***HL****: Hearing loss*
